# ‘Propped and prone’ positioning reduces respiratory events in spontaneously breathing preterm infants: A randomised triple crossover study

**DOI:** 10.1111/jpc.16241

**Published:** 2022-10-31

**Authors:** Christopher M Richmond, Fabian Ring, Lacey Richmond, Erika Rossouw, Emma Ballard, Pita Birch

**Affiliations:** ^1^ Newborn Care Unit Gold Coast University Hospital Gold Coast Queensland Australia; ^2^ Genetic Health Queensland Royal Brisbane & Women's Hospital Brisbane Queensland Australia; ^3^ School of Medicine Griffith University Gold Coast Queensland Australia; ^4^ School of Nursing & Midwifery University of Western Sydney Penrith New South Wales Australia; ^5^ QIMR Berghofer Medical Research Institute Brisbane Queensland Australia; ^6^ School of Nursing Midwifery and Social Work University of Queensland Brisbane Queensland Australia; ^7^ Department of Neonatology Mater Mother's Hospitals South Brisbane Queensland Australia

**Keywords:** apnoea, bradycardia, enteral feeding, neonatal health, preterm infants, prone position

## Abstract

**Aim:**

We compared effects of infant positioning and feed‐rate interventions on respiratory events and oximetry parameters in spontaneously breathing preterm infants born <32 weeks gestation managed in a neonatal unit.

**Methods:**

A randomised triple crossover design was employed. *n* = 68 infants underwent three test conditions A: control (supine/flat, gravity bolus feeds), B: position intervention (propped/prone) and C: feed‐rate intervention (continuous pump feeds) in randomised sequence over three consecutive days. Primary outcomes were number of events (apnoea, bradycardia and desaturation) and percentage time SpO_2_ < 80% over 24 h. The secondary outcome was percentage time SpO_2_ ≥ 88%. Treatment effects were estimated using linear mixed‐effects models.

**Results:**

Propped/prone positioning significantly reduced events and improved percentage time SpO_2_ < 80% and ≥88% compared to both other conditions (all *P* < 0.001). Outcomes for the feed‐rate intervention were not significantly different to control.

**Conclusions:**

Alternative infant positioning should be considered in preterm infants managed in the neonatal unit.

## What is already known on this topic

1. Cardiorespiratory events are common in preterm infants and reducing such events is a cornerstone of neonatal care.
2. Position and feed‐rate interventions are frequently utilised without a clear evidence base.
3. To our knowledge no studies have evaluated such interventions during or after feeds in spontaneously breathing infants prior to introduction of oral feeds.

## What this paper adds

1. This study evaluates the impact of feeding interventions (positional and feed‐rate) on cardiorespiratory events in spontaneously breathing preterm infants born <32 weeks gestation.
2. Propped/prone positioning reduces events and improves oximetry profile compared with both neutral position and continuous pump feeds.
3. Position interventions should be considered in developing neonatal care plans for the late preterm infant.

Cardiorespiratory events are common in preterm infants and temporal relationships with feeding often prompt positional or feed‐rate interventions. Apnoea of prematurity is a pause in breathing of more than 15–20 s, with or without accompanying oxygen desaturation and/or bradycardia in infants born less than 37 weeks gestation.^1^, ^2^, ^3^ It occurs in nearly all infants born less than 29 weeks gestation, in 54% between 30 and 31 weeks, and 15% between 32 and 33 weeks.^4^ Proposed mechanisms for feed‐related apnoea include gastric distension (reducing functional residual capacity) and upper airway changes.^5^ Prolonged apnoea may impair tissue perfusion and has been implicated in adverse neurodevelopmental outcomes.^6^ Reducing respiratory events is a cornerstone of neonatal care, frequently through use of invasive and non‐invasive respiratory support^7^ and caffeine/methylxanthines.^8^

Prone positioning may improve cardiopulmonary stability and respiratory dynamics^9^ and reduce gastric post‐feed residuals,^10^ feed intolerance and gastro‐oesophageal reflux.^11^ Positive effects on sleep^12^ and neurodevelopment^13^ have also been demonstrated. Prone positioning improves oxygenation in preterm infants receiving mechanical ventilation^14^, ^15^ but has not been shown to reduce apnoea in spontaneously breathing preterm infants.^16^, ^17^ Apnoea reduction has been demonstrated with Kangaroo mother care^18^ and semi‐elevated side‐lying^19^ in older preterm infants receiving oral/suck feeds. No studies have addressed positional interventions during or after feeds in the spontaneously breathing infant prior to introduction of oral feeds.

A 2021 Cochrane systematic review examining pump versus gravity feeds identified a single small crossover trial, precluding meta‐analysis,^20^ with a non‐significant trend towards increased respiratory rate following push gavage/pump feeds in *n* = 31 infants.^21^ A Cochrane meta‐analysis of seven trials found no difference in respiratory events in infants receiving nasogastric bolus versus continuous feeds.^22^ One study suggested a trend towards increased apnoea with continuous feeds.^23^ A subsequent crossover trial (*n* = 33) associated continuous feeding with increased apnoea and hypoxia.^5^ No studies were identified examining the effect of slower continuous pump feeds in spontaneously breathing preterm infants.

This study evaluates the impact of feeding interventions (positional and feed‐rate) on respiratory event frequency in spontaneously breathing preterm infants born less than 32 weeks' gestation managed in a neonatal unit, prior to introduction of nutritive oral feeds. We evaluate whether alternative infant and cot positioning (supine vs. ‘propped and prone’) and slowing of feed‐rate (gravity feeds vs. pump) reduce frequency of apnoea and/or improve oximetry profiles.

## Methods

**Fig. 1:**
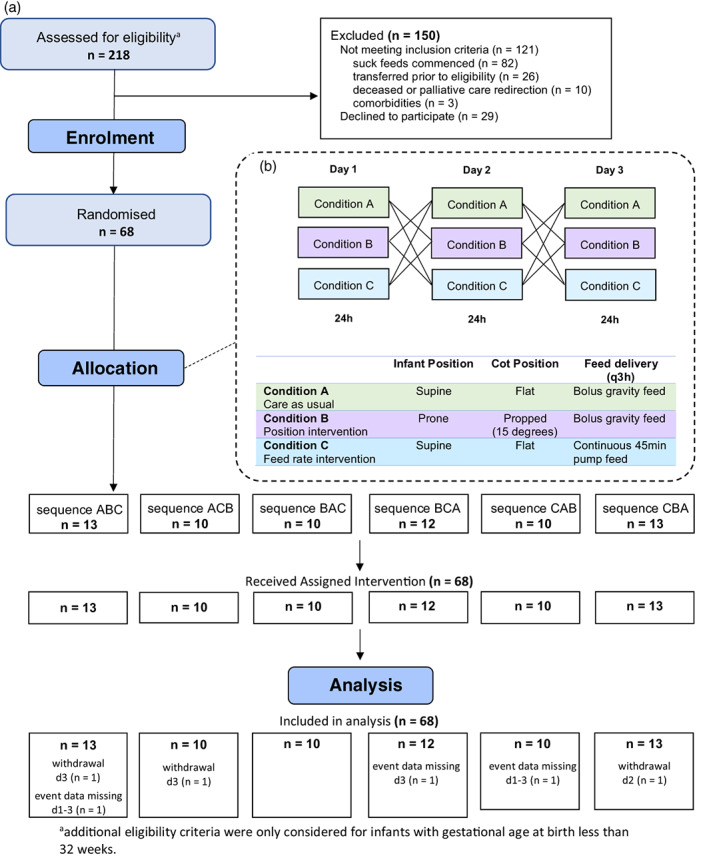
(a) CONSORT flowchart and (b) conditions and sequences for randomised triple crossover design. ^a^Additional eligibility criteria were only considered for infants with gestational age at birth less than 32 weeks.

Randomised triple crossover design was employed whereby each infant underwent three test conditions in randomised sequence, each over a 24‐h period for three consecutive days without washout (Fig. 1). Test conditions compared were A: usual care (supine in flat cot; usual nasogastric gravity bolus feeds); B: position intervention (prone with cot/isolette propped at 15° continuously for the 24‐h period; usual gravity bolus feeds) and C: feed‐rate intervention (supine in flat cot; pump feeds over 45 min). Infants were randomly assigned to one of six groups, defined by sequence permutations of the test conditions (ABC; ACB; BCA; BAC; CAB and CBA). The conditions represented nursing approaches already employed in this and other units. The study permitted control of conditions to facilitate meaningful comparison and guide practice.

### Study population

We recruited 68 preterm infants (birth gestation < 32 weeks) from an Australian tertiary neonatal unit. Eligible infants were: (i) self‐ventilating for at least 5 days prior to commencement; (ii) receiving full enteral feeds (>150 mL/kg/day) for at least 5 days and third‐hourly feeding for at least 48 h and (iii) not yet receiving formal nutritive oral/suck feeds. This group was selected to minimise confounding variables, such as changes to ventilatory parameters or enteral feed volumes during the study period. Infants receiving low‐flow sub‐nasal oxygen (<0.5 L/min) were eligible provided other respiratory criteria were met. Infants receiving caffeine citrate were included; however, dose was only adjusted for body weight during the trial period. Infants receiving nutritive oral feeds (counted towards daily fluid quota) were excluded as breast/bottle‐feeding precluded control of test conditions. All infants meeting criteria were considered for recruitment, irrespective of baseline respiratory events.

Infants with significant comorbidities were excluded, including those that might preclude alternative positioning (surgical indications, symptomatic congenital heart disease) or confound interpretation of outcome measures (congenital upper airway obstruction, pharmacotherapy for gastro‐oesophageal reflux). Additional exclusion criteria included palliative care redirection or imminent discharge/transfer.

### Outcome measures

Primary study outcomes were: (i) number of respiratory events and (ii) percentage of time with SpO_2_ less than 80%, both defined over a 24‐h period. A secondary outcome was percentage of time with SpO_2_ greater than or equal to 88% over 24 h. A respiratory ‘event’ was defined as one or a combination of clinically significant apnoea, desaturation or bradycardia. Apnoea was considered a pause in breathing of at least 15 s, or when accompanied by desaturation and/or bradycardia. Desaturation was defined as peripheral capillary oxygen saturation less than 80% for at least 5 s. Bradycardia represented heartrate less than 100 bpm for at least 5 s. Coincident apnoea, bradycardia and/or desaturation were counted as one event. Events were measured using the Philips Patient Information Center iX monitoring station (Philips, Eindhoven, Netherlands) with standard leads, recording traces of heartrate, respirations and SpO_2_ with 5 s averaging time.

Manual review of event triggers was undertaken by four investigators who were blinded to the condition sequence using standardised data collection sheets. Events were reconciled with dedicated nursing observation records and counted based on clinical assessment. Oximetry histogram generation by the monitoring system occurred at the conclusion of each 24‐h study period.

Baseline demographic data were recorded including gender, twin status, gestational age and weight (birth and commencement), caffeine administration and supplemental oxygen to describe our cohort. Data were recorded in an encrypted Microsoft Excel spreadsheet.

### Position intervention

Infants undertaking Condition A (‘supine and flat’) were placed supine with the cot in neutral (horizontal) position. The positional intervention, Condition B (‘propped and prone’), combined infant and cot position, with infants placed prone with head turned to one side and cot inclined at 15° (default cot elevation achievable within the unit). Positioning during Conditions A and B was maintained as default position throughout each 24‐h test period, but allowed for position change for routine nursing and medical care. Parent handling (holding and skin‐to‐skin) was encouraged, however avoided during and within 1 h of feeding.

### Feed‐rate intervention

Infants in Conditions A and B received usual third‐hourly gravity bolus feeds via nasogastric tube, typically over 5–15 min. The feed‐rate intervention (Condition C) was defined by third‐hourly feed volume given over 45 min by pump, with usual supine/flat infant position. Pump feeds were administered via Vygon (Écouen, France) Nutrisafe‐2 pump and giving set.

### Sample size

We aimed to recruit 90 infants to detect a 40% reduction in the log(number of events) and a 50% reduction in the log(percentage time with SpO_2_ < 80%) for a two‐sided test with 80% power and 5% alpha (additional information provided in Appendix S1).

### Statistical analysis

Additional statistical methodology is provided in Appendix S1. Data were analysed using STATA v15.1 (TX). Differences between sequence and demographics were examined using Fisher's exact test (categorical data) and one‐way ANOVA or Kruskal–Wallis test (continuous data). Number of events was examined using a generalised linear mixed effects model (GL(M)M) assuming negative binomial family and log link (Appendix S1). Linear mixed effects modelling (LMM) estimated treatment effect with the logit response for each oximetry outcome. All models contained sequence, Day and Condition as fixed effects and patient as a random effect. Marginal mean estimates for each treatment and corresponding 95% confidence intervals (CIs), holding all other variables at their means, were reported. For the LMM, back‐transformed marginal means and their CIs were reported. Incidence rate ratios (IRRs) and 95% CIs were reported for the GL(M)M. Both the modified intention‐to‐treat (mITT, all randomised patients with at least one evaluation pertaining to outcome of interest) and per‐protocol populations (PPs) were analysed.

## Results

### Baseline participant characteristics

Of 68 infants included in the study, 23 (34%) were twins and 38 (56%) were male (Table 1). One twin pair was enrolled in the same treatment sequence (BAC). Mean commencement corrected gestation was 34.6 weeks (SD 1.2) and median weight 2023 g (IQR 1908–2350). Twenty‐six infants (38%) received supplemental oxygen and 19 received caffeine (28%). Although not statistically significant, infants in sequence BAC appeared to be younger compared to other sequences, however sequences were otherwise similar for gestation, weight and support.

**Table 1:** Patient characteristics; overall and by treatment sequence (*n* = 68)

Characteristic	Statistic	Overall	Treatment sequence						*P* value
			ABC	ACB	BAC	BCA	CAB	CBA	
			*n* = 13	*n* = 10	*n* = 10	*n* = 12	*n* = 10	*n* = 13	
Gestational age at birth (weeks)	Mean (SD)	29.0 (1.9)	29.3 (2.2)	28.0 (2.0)	29.7 (1.7)	29.0 (1.8)	29.0 (2.1)	29.2 (1.8)	0.48
Corrected gestational age Day 1 (weeks)	Mean (SD)	34.6 (1.2)	34.8 (1.4)	34.8 (1.6)	34.0 (1.3)	34.6 (1.3)	34.3 (0.9)	34.8 (0.9)	0.60
Birthweight (g)	Mean (SD)	1276 (367)	1300 (391)	1101 (335)	1391 (307)	1290 (369)	1253 (407)	1305 (394)	0.65
Current weight Day 1 (g)	Median (IQR)	2023 (1908–2350)	2035 (1965–2385)	1990 (1855–2369)	1888 (1755–2225)	2023 (1968–2560)	2180 (1925–2310)	2050 (1960–2310)	0.49
Gender (male)	*n* (%)	38 (55.9%)	9 (69.2%)	6 (60.0%)	5 (50.0%)	8 (66.7%)	4 (40.0%)	6 (46.2%)	0.69
Twins	*n* (%)	23 (33.8%)	3 (23.1%)	5 (50.0%)	5 (50.0%)†	4 (33.3%)	4 (40.0%)	2 (15.4%)	0.40
Supplemental O_2_ during study	*n* (%)	26 (38.2%)	5 (38.5%)	6 (60.0%)	2 (20.0%)	5 (41.7%)	3 (30.0%)	5 (38.5%)	0.62
O_2_ flow rate (*n* = 25, L/min)	Median (IQR)	0.05 (0.05–0.10)	0.09 (0.05–0.21)	0.08 (0.03–0.20)	0.07 (0.03–0.10)	0.05 (0.05–0.10)	0.05 (0.03–0.20)	0.05 (0.05–0.10)	0.91
Caffeine during study period	*n* (%)	19 (27.9%)	1 (7.7%)	2 (20.0%)	5 (50.0%)	3 (25.0%)	5 (50.0%)	3 (23.1%)	0.17

† Pair of twins allocated to same condition sequence.

Ten data points were missing for daily number of events (5%) and there were four missing data points each for time SpO_2_ < 80% and time SpO_2_ ≥ 88% (2%). These were attributable to monitor retrieval failure (two infants; ABC and CAB), study withdrawal due to introduction of oral feeds during the study period (two infants; ABC and CBA) or clinical deterioration requiring respiratory support (one infant; ACB) (Fig. 1).

### Outcomes

Condition B (positional intervention) demonstrated lower number of events, smaller percentage time with SpO_2_ < 80% and greater percentage time with SpO_2_ ≥ 88% compared to the other two treatments (Table 2). In all models, Conditions A and C were not significantly different and Condition B was significantly different to both treatments (all *P* < 0.001, Table S3). Condition B lowered frequency of events by 50% (IRR 0.50 [95% CI 0.41–0.62]) compared to Condition A and by 42% (IRR 0.58 [95% CI 0.38–0.80]) compared to Condition C. Percentage time with SpO_2_ < 80% for Condition B is smaller by 0.3 units (50% lower) than Conditions A/C. In a 24‐h period, this equates to a change from 8.64 min with SpO_2_ < 80% with Conditions A/C to 4.32 min with Condition B (Table 2).

**Table 2:** Marginal mean estimates for number of events, percentage of time spent with SpO_2_ less than 80% and 88% or more for each treatment

Treatment	Number of events	% of time with SpO_2_ < 80%	% of time with SpO_2_ ≥ 88%
	Mean (95% CI)	Mean (95% CI)†	Mean (95% CI)†
A: Care as usual	27.7 (19.1–36.3)^a^	0.6 (0.5–0.8)^a^	97.8 (97.0–98.3)^a^
B: Positional intervention	13.8 (9.4–18.3)^ab^	0.3 (0.2–0.4)^ab^	98.8 (98.4–99.0)^ab^
C: Feed rate intervention	24.9 (17.2–32.7)^b^	0.6 (0.4–0.8)^b^	97.8 (97.0–98.3)^b^

^ab^Margins sharing a letter are significantly different at the 5% level.

† Back transformed estimates and 95% CIs.

There was a statistically significant increase in percentage time with SpO_2_ ≥ 88% with Conditions B compared to Conditions A/C; however, this difference may not be clinically significant, increasing an already high proportion from 97.8 to 98.8% (additional 14.4 min in this favourable oximetry range over 24 h).

**Fig. 2:**
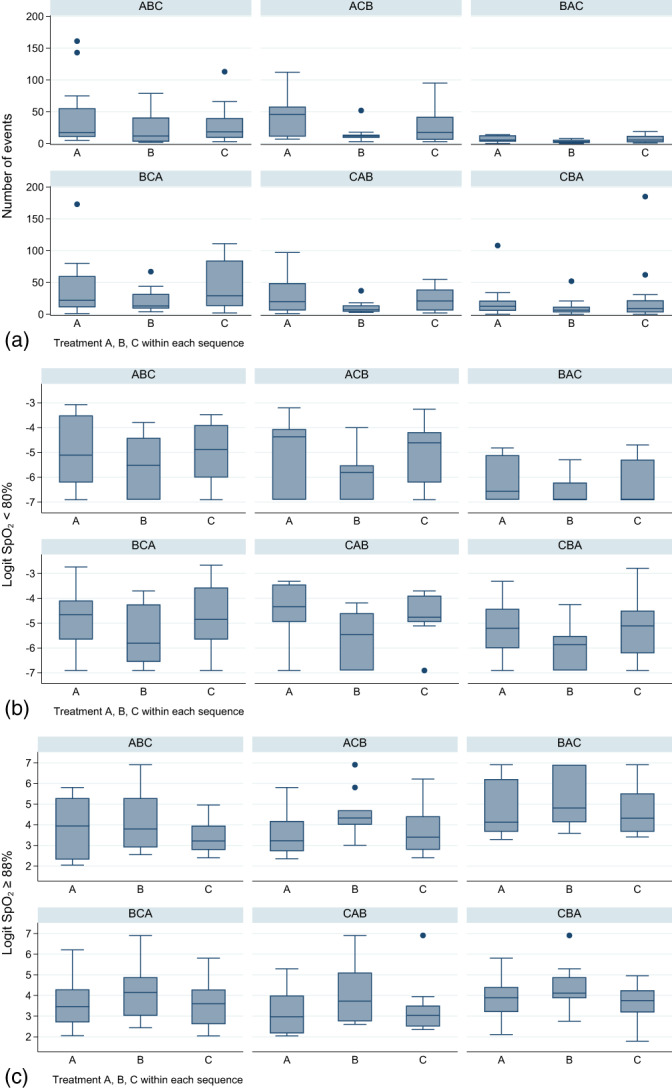
Box plots of (a) number of events (b) logit transformed percentage of time with peripheral oxygen saturation less than 80% and (c) logit transformed percentage of time spent with peripheral oxygen saturation 88% or more; presented in treatment order of (a), (b) and (c) for each sequence. The first letter in the treatment sequence corresponds to the treatment on day 1, the second letter treatment on day 2 and the third letter on day 3.

Descriptive statistics for event frequency and oximetry by day and sequence are given in Table S1. Treatment sequence was significant for the number of events model but not oximetry models (Table S3). Sequence BAC appeared to have fewer events, smaller percentage time with SpO_2_ < 80% and greater percentage time with SpO_2_ ≥ 88% compared to other sequences (consistent across days). The second treatment sequence commencing with Condition B (BCA) was more similar to the other sequences than to BAC (Fig. 2). Other than two twins being randomised to BAC, there were no other infant characteristics unique to the group (Table 1). Day 1 analysis is given in Table S2, supporting modelled findings described below.

No carryover effect was present in any models evaluated. Two outliers (StudyID 20, day 1 and StudyID 52, day 2) were present in sequence BAC for number of events, identified on scatterplot of deviance by treatment sequence; however, these were not present on Pearson plots and their exclusion did not significantly change treatment estimates or between‐infant variance. The relationships and size of effects did not change when LMM was run using the log(number of events) compared to (GL(M)M). Similar results were obtained for both miTT and PP data sets for all outcomes (Tables S4–S6).

### Summary

The positional intervention (Condition B) was superior compared to other conditions, significantly reducing number of events and percentage of time with SpO_2_ < 80% and increasing percentage of time with SpO_2_ ≥ 88%. Event frequency and percentage time with SpO_2_ < 80% and ≥88% did not differ significantly between the usual care (Condition A) and feed‐rate intervention (Condition C).

## Discussion

‘Propped and prone’ positioning is superior to both supine/flat positioning and continuous pump‐feeding in reducing respiratory events and percentage time with SpO_2_ < 80%, and increasing percentage time with SpO_2_ ≥ 88% in late‐preterm infants. Previous studies have demonstrated event reduction in younger, mechanically ventilated infants^16^, ^17^ and those receiving oral/suck feeds.^19^, ^24^ To our knowledge, this is the first study demonstrating reduced apnoea and desaturation with propped/prone positioning in spontaneously breathing infants prior to oral‐feed introduction, with previous meta‐analysis (five studies, *n* = 114) having insufficient evidence to determine an effect.^16^ The reduction in events was statistically and clinically significant, with alternative positioning halving events compared to supine/flat positioning, and lowering by 42% compared to feed‐rate intervention alone. These differences are clinically meaningful equating to propped/prone positioning reducing time spent with SpO_2_ < 80% by 4.3 min in a 24‐h period.

There were no differences in primary or secondary outcomes between feed‐rate intervention (continuous pump‐feeds) and care‐as‐usual (gravity bolus feeds). This result is consistent with previous limited reviews^20^, ^22^; however, we did not identify a trend towards increased apnoea/events with continuous feeds proposed in some studies.^5^, ^23^

### Limitations

There was <5% missing data for primary and secondary outcomes, some missing at random and some not. The sequence effect in the events model is thought to be due to patient characteristics. Sensitivity analysis showed results are robust and confirm Condition B as superior.

Our position intervention combined both infant and cot position, as these typically occur together and their separation would complicate crossover design. Further study is required to evaluate any separate contributions. Prone positioning improves quiet sleep, which is known to positively impact frequency of respiratory events.^15^ We did not measure or control for sleep state, thus it was not possible to determine the relative impact of duration of active and quiet sleep on observed differences.

Additionally, infants undertook interventions for the entire 24‐h period. Developmental implications of this approach require further evaluation, and additional studies could evaluate if interventions during feed times only might preserve this effect. This study targeted ‘stable, growing’ spontaneously breathing preterm infants (mean gestation of 34.6 weeks). Results may not, therefore, be applicable to younger infants or those receiving additional respiratory supports. These findings may also conflict with recommendations to promote supine positioning as infants approach discharge to reduce the incidence of Sudden Infant Death Syndrome and must therefore consider individual infant factors and disposition planning.

Our proposed sample size was not achieved for a number of reasons, most importantly premature cessation of the study to the Covid‐19 pandemic. Recruitment was additionally impacted by unexpectedly short eligibility windows between cessation of respiratory support and oral‐feed introduction. We observed, however, smaller between‐ and within‐subject variances and an increased percent reduction in number of events than predicted by the pilot (50% compared with 40% from the pilot). Post hoc power analyses showed an *n* = 36 sample was sufficient with the modelled variance estimates for number of events to achieve 80% power.

### Conclusions

Propped and prone positioning should be considered by neonatal physicians and nurses caring for spontaneously breathing, late‐preterm infants with established enteral feeds, particularly those in whom respiratory events have been problematic. There does not appear to be evidence to support feed‐rate interventions in this group for this indication. Individual consideration must necessarily be given to infant factors, nursing cares, medical interventions and parental preference. Position interventions must also be balanced with safe sleeping considerations as infants approach hospital discharge. Although it is not feasible to maintain a single infant position for all cares and interventions, neonatal units might consider prioritisation of propped/prone positioning in developing infant care plans. Larger, multi‐centre studies are required to further evaluate this effect and permit further subgroup analyses.

## Author Contributions

Conceptualization & Methodology: Christopher M Richmond and Pita Birch; Data Collection: Christopher M Richmond, Fabian Ring, Lacey Richmond, and Erika Rossouw, Formal Analysis: Christopher M Richmond and Emma Ballard; Validation: Christopher M Richmond, Emma Ballard, Pita Birch; Supervision: Pita Birch; Writing, original draft: Christopher M Richmond, and Emma Ballard; Writing, review & editing: Christopher M Richmond, Fabian Ring, Lacey Richmond, Erika Rossouw, Emma Ballard, and Pita Birch.

## Ethics Statement

This project received Human Research Ethics Committee approval (HREC/15/QGC/297) and site‐specific approval (SSA/15/QGC/300) through Gold Coast Health (Southport, Queensland, Australia) in 2016.

## Supporting information


**Appendix S1** Supporting Information

## Data Availability

Data analysed are supplied in Supporting Information and are also available upon request to the corresponding authors.
