# Supplementary material for: ‘Propped and prone’ positioning reduces respiratory events in spontaneously breathing preterm infants: A randomised triple crossover study
Source: J Paediatr Child Health. 2022 Oct 31;59(1):81–8. doi: 10.1111/jpc.16241 (PMC10092656; doi:10.1111/jpc.16241)
Supplement: Supplementary file 1 — Appendix S1 Supporting Information [file JPC-59-81-s001.docx]

**Supplementary Methods**

*Prestudy Design, Sample size*

A pre-study audit of n=10 infants receiving standard care (equivalent to Condition A) permitted estimates of between-subject and within-subject variance for primary outcomes. We aimed to recruit 90 infants to detect a 40% reduction in the log(number of events), with correlation between observations within the same subject of 0.6, and a 50% reduction in the log(percentage time with SpO2 <80%), with correlation between observations within the same subject of 0.4 for a two-sided test with 80% power and 5% alpha.

*Study Design*

We employed a randomised triple crossover design in which n = 68 infants each underwent three test conditions, in randomised sequence, each over 3x 24-hour period without washout (Figure 1). Test conditions were:

- A: usual care (infant nursed supine in a flat cot with third hourly gravity bolus feeds);
- B: position intervention (nursed prone with cot or isolette propped at 15 degrees continuously for the 24-hour period, with usual third-hourly gravity bolus feeds) and
- C: feed rate intervention (nursed supine in a flat cot with pump feed administered over 45 minutes).

Informed written consent was obtained from caregivers, and infants were randomly assigned to one of six groups, with sequence defined by computer-assisted random number generation of test condition sequence permutations (ABC;ACB;BCA;BAC;CAB;CBA). Allocation concealment was achieved by use of sealed study envelopes. Recruitment occurred between July 2016 and January 2020.

*Data Analysis*

Data were analysed using STATA v15.1 (Texas, USA). Subject demographics were described using frequency and percentage for categorical variables, and continuous variables by mean and standard deviation or median and interquartile range (IQR) if not normally distributed. Differences between sequence and demographics were examined using Fisher’s exact test (categorical data) and one-way ANOVA or Kruskal-Wallis test (continuous data).

Number of events was examined using a generalised linear mixed effects model (GL(M)M) assuming negative binomial family and log link. Due to truncation of oxygen saturation percentage scales and skewed distribution, SpO2 <80% values of 0 were changed to 0.1 and data logit transformed prior to analysis using the formula: $\mathrm{logit}\left( SpO2lt80 \right)=\ln\left( SpO2lt80\_adj/(100-SpO2lt80\_adj \right))$. For SpO2 ≥88%, values of 100 were changed to 99.9 and data logit transformed. Linear mixed effects modelling (LMM) estimated treatment effect with the logit response for each respiratory outcome.

For each outcome the interaction between day and treatment was examined using their interaction as a fixed effect. Carryover effect was examined using dummy variables to describe the preceding treatment. All final models contained sequence (Group), period (Day) and Condition as fixed effects and patient (StudyID) as a random effect. Restricted maximum likelihood estimates (REML) were reported, residuals examined and homoscedasticity verified. Marginal mean estimates for each treatment and corresponding 95% confidence intervals (CIs), holding all other variables at their means, were reported. For the LMM, back-transformed marginal means and their CIs were reported. Incidence rate ratios and 95% CIs were reported for the GL(M)M. Both the modified intention-to-treat population (mITT, all randomised patients with at least one evaluation pertaining to the outcome of interest) and per-protocol population (PP) were analysed.

*Effect Validation*

A sequence effect was observed during modelling of the number of events. Univariate analysis was conducted to explore whether there were patient characteristics that made sequence BAC unique, whether sequence effects were consistent across days and whether they affected other outcomes. Table S1 shows that the number of events is significantly different between sequences on day 1 and borderline significant on days 2 and 3. SpO2 less than 80% is borderline significantly different on days 1 and 2. SpO2 88% or more is borderline significant on day 1. Table S2 shows the comparison of treatments on day 1 only using the Kruskal-Wallis test and one-way ANOVA. Treatment B was found to have significantly fewer events than Treatment A however there was no significant differences between Treatment B and C or A and C. Table S3 shows the final model parameter estimates for each outcome. Table S4 describes additional modelling where a logarithm transformation (ln(NoEvents+1)) was applied to the number of events which were then examined with a linear mixed effects model (LMM), as originally proposed with sample size calculation beside the GL(M)M model. Analysis was completed for both mITT and PP populations. Tables S5 and S6 compare SpO2 outcomes using LMM on mITT and PP populations.

*Clinical Trial Registration*

WHO Universal Trial Number (UTN): U1111-1277-2557

ANZCTR: ACTRN12622000672752

This trial was retrospectively registered with ANZCTR. This study followed an initial project (audit) which generated a subsequent detailed project proposal. A detailed statistical analysis plan and protocol was in place prior to commencement of the project, however clinical trial registration was not undertaken prospectively. There was intent to publish irrespective of whether a significant difference was observed.

**Table S1:** Descriptive statistics for the number of events, percentage of time spent with SpO2 less than 80% and 88% or more by day and treatment sequence.

^abcd^values sharing a letter are significantly different at the 5% level.

| **Outcome** | **Statistic** | **Overall** | **Treatment sequence** | | | | | | **p-value** |
| --- | --- | --- | --- | --- | --- | --- | --- | --- | --- |
|  |  |  | **ABC** | **ACB** | **BAC** | **BCA** | **CAB** | **CBA** |  |
|  |  |  | n=13 | n=10 | n=10 | n=12 | n=10 | n=13 |  |
| **Day 1** |  |  |  |  |  |  |  |  |  |
| Number of events | missing n | 2 | 1 | 0 | 0 | 0 | 1 | 0 |  |
|  | n | 66 | 12 | 10 | 10 | 12 | 9 | 13 |  |
|  | median (IQR) | 13 (5 - 37) | 17 (11 - 56)^a^ | 46 (11 - 58)^b^ | 3 (1-6)^abcd^ | 13 (9 - 32)^c^ | 21 (6 - 39)^d^ | 9 (3 - 22) | 0.001 |
| % of time with SpO2 < 80% | missing n | 0 | 0 | 0 | 0 | 0 | 0 | 0 |  |
|  | n | 68 | 13 | 10 | 10 | 12 | 10 | 13 |  |
|  | median (IQR) | 0.6 (0.1 - 1.8) | 0.6 (0.2 - 2.9)^a^ | 1.3 (0.1 - 1.7) | 0.1 (0.0 - 0.2)^ab^ | 0.3 (0.2 - 1.5) | 0.9 (0.7 – 2.0)^b^ | 0.6 (0.2 - 1.1) | 0.026 |
| % of time with SpO2 ≥ 88% | missing n | 0 | 0 | 0 | 0 | 0 | 0 | 0 |  |
|  | n | 68 | 13 | 10 | 10 | 12 | 10 | 13 |  |
|  | median (IQR) | 98.0 (94.6 - 99.2) | 98.1 (91.0 - 99.5) | 96.2 (93.8 - 98.5) | 99.1 (98.4 - 99.9)^a^ | 98.4 (95.3 - 99.3) | 95.4 (92.4 - 97.1)^a^ | 97.7 (96.0 - 98.6) | 0.034 |
| **Day 2** |  |  |  |  |  |  |  |  |  |
| Number of events | missing n | 3 | 1 | 0 | 0 | 0 | 1 | 1 |  |
|  | n | 65 | 12 | 10 | 10 | 12 | 9 | 12 |  |
|  | median (IQR) | 11 (5 - 35) | 12 (3 -41) | 18 (6 - 42) | 6 (3 - 13)^a^ | 29 (13 - 85)^ab^ | 20 (6 - 49) | 7 (3-12)^b^ | 0.024 |
| % of time with SpO2 < 80% | missing n | 0 | 0 | 0 | 0 | 0 | 0 | 0 |  |
|  | n | 67 | 13 | 10 | 10 | 12 | 10 | 12 |  |
|  | median (IQR) | 0.5 (0.1 - 1.4) | 0.4 (0.1 - 1.2) | 1.0 (0.2 - 1.5) | 0.2 (0.1 - 0.6) | 0.9 (0.4 - 2.8) | 1.3 (0.7 - 3.1) | 0.3 (0.1 - 0.4) | 0.047 |
| % of time with SpO2 ≥ 88% | missing n | 0 | 0 | 0 | 0 | 0 | 0 | 0 |  |
|  | n | 67 | 13 | 10 | 10 | 12 | 10 | 12 |  |
|  | median (IQR) | 97.8 (94.8 - 98.9) | 97.8 (94.8 - 99.5) | 96.8 (94.2 - 98.8) | 98.4 (97.5 - 99.8) | 97.3 (93.1 - 98.7) | 95.1 (89.1 - 98.2) | 98.4 (98.0 - 99.3) | 0.087 |
| **Day 3** |  |  |  |  |  |  |  |  |  |
| Number of events | missing n | 5 | 1 | 1 | 0 | 1 | 1 | 1 |  |
|  | n | 63 | 12 | 9 | 10 | 11 | 9 | 12 |  |
|  | median (IQR) | 12 (5 - 19) | 19 (9 - 40) | 12 (9 - 14) | 6 (2 - 12)^a^ | 22 (11 - 60)^a^ | 7 (4 - 14) | 13 (6 - 22) | 0.039 |
| % of time with SpO2 < 80% | missing n | 3 | 1 | 1 | 0 | 0 | 0 | 1 |  |
|  | n | 65 | 12 | 9 | 10 | 12 | 10 | 12 |  |
|  | median (IQR) | 0.5 (0.1 - 1.0) | 0.8 (0.3 - 2.0) | 0.3 (0.1 - 0.4) | 0.1 (0.0 - 0.5) | 1.0 (0.4 - 1.7) | 0.5 (0.1 - 1.0) | 0.6 (0.3 - 1.2) | 0.070 |
| % of time with SpO2 ≥ 88% | missing n | 3 | 1 | 1 | 0 | 0 | 0 | 1 |  |
|  | n | 65 | 12 | 9 | 10 | 12 | 10 | 12 |  |
|  | median (IQR) | 97.8 (95.3 - 99.1) | 96.1 (94.2 - 98.0) | 98.7 (98.2 - 99.1) | 98.6 (97.5 - 99.6) | 97.0 (93.7 - 98.6) | 97.7 (94.0 - 99.4) | 98 (96.1 - 98.8) | 0.064 |

**Table S2:** Comparison of treatments on day one only.

| **Period** | **Treatment** | **n** | **No. of events** | |
| --- | --- | --- | --- | --- |
|  |  |  | **Raw data** | **Ln transformed data** |
|  |  |  | median (IQR) | mean (95% CI) |
| Day 1 only | A | 22 | 26 (11 - 58)^a^ | 3.3 (2.9 - 3.8)^a^ |
|  | B | 22 | 8 (3 - 13)^a^ | 2.1 (1.6 - 2.6)^a^ |
|  | C | 22 | 17 (3 - 31) | 2.6 (2.0 - 3.1) |
| p-value |  |  | 0.006 | 0.003 |

^a^ Medians sharing a letter are significantly different at the 5% level.

**Table S3:** Parameter estimates for the generalised linear mixed effects model for the number of events and linear mixed effects model models for Logit % of time with SpO2 < 80% and SpO2 ≥ 88%.

|  | **GL(M)M: Number of events** | | | | | **LMM: Logit % of time with SpO2 < 80%** | | | | | **LMM: Logit % of time with SpO2 ≥ 88%** | | | | |
| --- | --- | --- | --- | --- | --- | --- | --- | --- | --- | --- | --- | --- | --- | --- | --- |
|  | **IRR** | **SE** | **95% CI** | **z** | **p-value** | **Beta** | **SE** | **95% CI** | **z** | **p-value** | **Beta** | **SE** | **95% CI** | **z** | **p-value** |
| Group |  |  |  |  | 0.001 |  |  |  |  | 0.083 |  |  |  |  | 0.146 |
| ABC | Ref |  |  |  |  | Ref |  |  |  |  | Ref |  |  |  |  |
| ACB | 1.07 | 0.45 | 0.47 - 2.45 | 0.16 | 0.87 | -0.12 | 0.46 | -1.02 - 0.78 | -0.26 | 0.80 | 0.11 | 0.48 | -0.83 - 1.05 | 0.23 | 0.81 |
| BAC | 0.24 | 0.10 | 0.10 - 0.57 | -3.28 | 0.001 | -1.10 | 0.46 | -2.00 - -0.20 | -2.39 | 0.017 | 1.06 | 0.48 | 0.12 - 1.99 | 2.21 | 0.027 |
| BCA | 1.34 | 0.54 | 0.61 - 2.96 | 0.73 | 0.47 | 0.14 | 0.44 | -0.72 - 1.00 | 0.31 | 0.76 | 0.00 | 0.46 | -0.89 - 0.89 | 0.00 | 1.00 |
| CAB | 0.77 | 0.34 | 0.33 - 1.83 | -0.58 | 0.56 | 0.17 | 0.46 | -0.73 - 1.07 | 0.37 | 0.71 | -0.27 | 0.48 | -1.21 - 0.66 | -0.57 | 0.57 |
| CBA | 0.47 | 0.19 | 0.21 - 1.04 | -1.86 | 0.062 | -0.33 | 0.43 | -1.17 - 0.51 | -0.77 | 0.44 | 0.18 | 0.45 | -0.70 - 1.05 | 0.40 | 0.69 |
| Treatment |  |  |  |  | <0.001 |  |  |  |  | <0.001 |  |  |  |  | <0.001 |
| A | Ref |  |  |  |  | Ref |  |  |  |  | Ref |  |  |  |  |
| B | 0.50 | 0.05 | 0.41 - 0.62 | -6.44 | <0.001 | -0.68 | 0.10 | -0.87 - -0.49 | -6.86 | <0.001 | 0.61 | 0.10 | 0.42 - 0.81 | 6.28 | <0.001 |
| C | 0.90 | 0.09 | 0.74 - 1.10 | -1.02 | 0.31 | -0.03 | 0.10 | -0.23 - 0.16 | -0.33 | 0.75 | 0.00 | 0.10 | -0.19 - 0.19 | -0.02 | 0.99 |
| Day |  |  |  |  | 0.21 |  |  |  |  | 0.11 |  |  |  |  | 0.72 |
| 1 | Ref |  |  |  |  | Ref |  |  |  |  | Ref |  |  |  |  |
| 2 | 0.93 | 0.10 | 0.76 - 1.45 | -0.66 | 0.51 | -0.01 | 0.10 | -0.20 - 0.19 | -0.07 | 0.94 | 0.01 | 0.10 | -0.18 - 0.20 | 0.05 | 0.96 |
| 3 | 0.83 | 0.09 | 0.67 - 1.02 | -1.76 | 0.078 | -0.19 | 0.10 | -0.38 - 0.01 | -1.88 | 0.060 | 0.07 | 0.10 | -0.12 - 0.26 | 0.73 | 0.47 |
| Intercept | 27.24 | 8.09 | 15.22 - 48.7 | 11.13 | <0.001 | -4.86 | 0.31 | -5.47 - -4.24 | -15.49 | < 0.001 | 3.58 | 0.33 | 2.94 - 4.22 | 11.01 | < 0.001 |
| ln alpha | -1.40 | 0.17 | -1.74 - -1.08 |  |  | n/a | n/a | n/a | n/a | n/a | n/a | n/a | n/a | n/a | n/a |
|  |  |  |  |  |  |  |  |  |  |  |  |  |  |  |  |
| \| 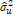 \| \| --- \| | 0.87 | 0.18 | 0.57 - 1.31 |  |  | 1.09 | 0.22 | 0.74 - 1.60 |  |  | 1.19 | 0.23 | 0.81 - 1.75 |  |  |
| 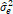 |  |  |  |  |  | 0.32 | 0.04 | 0.25 - 0.41 |  |  | 0.31 | 0.04 | 0.25 - 0.40 |  |  |

IRR incidence rate ratio, SE Standard error, CI confidence interval,
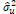
 between infant variance,
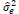
residual error

**Table S4:** Parameter estimates for the number of events using a linear mixed effects model and generalised linear mixed effects model models for the mITT and PP populations.

|  | **Modified intention-to-treat population** | | | | | **Per protocol population** | | | | |
| --- | --- | --- | --- | --- | --- | --- | --- | --- | --- | --- |
|  | n=194 observations, n=65 infants | | | | | n=189 observations, n=63 infants | | | | |
| **Parameter** | **Beta** | **SE** | **95% CI** | **z** | **p-value** | **Beta** | **SE** | **95% CI** | **z** | **p-value** |
| **LMM: Logarithm of number of events** | | | |  |  |  |  |  |  |  |
| Group |  |  |  |  |  |  |  |  |  |  |
| ABC | Ref |  |  |  |  | Ref |  |  |  |  |
| ACB | 0.03 | 0.41 | -0.78 - 0.84 | 0.07 | 0.94 | 0.05 | 0.42 | -0.77 - 0.86 | 0.11 | 0.91 |
| BAC | -1.31 | 0.41 | -2.11 - -0.50 | -3.18 | 0.001 | -1.31 | 0.40 | -2.10 - -0.52 | -3.24 | 0.001 |
| BCA | 0.23 | 0.39 | -0.53 - 1.00 | 0.60 | 0.55 | 0.18 | 0.39 | -0.59 - 0.96 | 0.47 | 0.64 |
| CAB | -0.27 | 0.42 | -1.10 - 0.56 | -0.64 | 0.52 | -0.27 | 0.42 | -1.08 - 0.54 | -0.65 | 0.52 |
| CBA | -0.74 | 0.39 | -1.50 - 0.01 | -1.92 | 0.054 | -0.58 | 0.38 | -1.33 - 0.17 | -1.51 | 0.13 |
| Treatment |  |  |  |  |  |  |  |  |  |  |
| A | Ref |  |  |  |  | Ref |  |  |  |  |
| B | -0.60 | 0.10 | -0.80 - -0.39 | -5.73 | <0.001 | -0.57 | 0.10 | -0.77 - -0.37 | -5.64 | <0.001 |
| C | -0.10 | 0.10 | -0.30 - 0.11 | -0.92 | 0.36 | -0.07 | 0.10 | -0.26 - 0.13 | -0.67 | 0.50 |
| Day |  |  |  |  |  |  |  |  |  |  |
| 1 | Ref |  |  |  |  | Ref |  |  |  |  |
| 2 | -0.08 | 0.10 | -0.28 - 0.12 | -0.75 | 0.45 | -0.09 | 0.10 | -0.28 - 0.11 | -0.85 | 0.40 |
| 3 | -0.15 | 0.10 | -0.35 - 0.05 | -1.44 | 0.15 | -0.16 | 0.10 | -0.35 - 0.04 | -1.56 | 0.12 |
| Intercept | 3.23 | 0.30 | 2.66 - 3.79 | 11.15 | <0.001 | 3.21 | 0.28 | 2.66 - 3.77 | 11.31 | <0.001 |
| \| 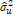 \| \| --- \| | 0.81 | 0.17 | 0.53 - 1.22 |  |  | 0.78 | 0.17 | 0.51 - 1.18 |  |  |
| \| 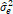 \| \| --- \| | 0.34 | 0.04 | 0.27 - 0.44 |  |  | 0.32 | 0.04 | 0.25 - 0.41 |  |  |
| **GL(M)M: Number of events** | | |  |  |  |  |  |  |  |  |
| Group |  |  |  |  |  |  |  |  |  |  |
| ABC | Ref |  |  |  |  | Ref |  |  |  |  |
| ACB | 0.07 | 0.42 | -0.76 - 0.89 | 0.16 | 0.87 | 0.05 | 0.43 | -0.79 - 0.89 | 0.11 | 0.91 |
| BAC | -1.41 | 0.43 | -2.26 - -0.57 | -3.28 | 0.001 | -1.41 | 0.43 | -2.25 - -0.58 | -3.32 | 0.001 |
| BCA | 0.29 | 0.40 | -0.50 - 1.01 | 0.73 | 0.47 | 0.22 | 0.41 | -0.58 - 1.02 | 0.54 | 0.59 |
| CAB | -0.26 | 0.44 | -1.11 - 0.60 | -0.58 | 0.56 | -0.26 | 0.43 | -1.10 - 0.59 | -0.59 | 0.56 |
| CBA | -0.75 | 0.40 | -1.54 - 0.04 | -1.86 | 0.062 | -0.61 | 0.40 | -1.40 - 0.18 | -1.52 | 0.13 |
| Treatment |  |  |  |  |  |  |  |  |  |  |
| A | Ref |  |  |  |  | Ref |  |  |  |  |
| B | -0.69 | 0.10 | -0.90 - -0.48 | -6.44 | <0.001 | -0.66 | 0.10 | -0.86 - -0.46 | -6.45 | <0.001 |
| C | -0.10 | 0.10 | -0.31 - 0.10 | -1.02 | 0.31 | -0.08 | 0.10 | -0.28 - 0.11 | -0.86 | 0.39 |
| Day |  |  |  |  |  |  |  |  |  |  |
| 1 | Ref |  |  |  |  | Ref |  |  |  |  |
| 2 | -0.07 | 0.10 | -0.27 - 0.14 | -0.66 | 0.51 | -0.08 | 0.10 | -0.28 - 0.12 | -0.79 | 0.43 |
| 3 | -0.19 | 0.10 | -0.39 - 0.02 | -1.76 | 0.078 | -0.19 | 0.10 | -0.39 - 0.00 | -1.92 | 0.055 |
| Intercept | 3.30 | 0.30 | 2.72 - 3.89 | 11.13 | <0.001 | 3.29 | 0.29 | 2.72 - 3.86 | 11.24 | <0.001 |
| 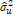ln alpha | -1.41 | 0.17 | -1.74 - -1.08 |  |  | -1.54 | 0.18 | -1.89 - -1.20 |  |  |
| \|  \| \| --- \| | 0.87 | 0.18 | 0.57 - 1.31 |  |  | 0.86 | 0.18 | 0.57 - 1.28 |  |  |

SE Standard error, CI confidence interval,
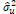
 between infant variance,
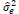
residual error

**Table S5:** Parameter estimates for the percentage of time spent with SpO2 less than 80% using LMM on mITT and PP populations.

|  | **Modified intention-to-treat population** | | | | | **Per protocol population** | | | | |
| --- | --- | --- | --- | --- | --- | --- | --- | --- | --- | --- |
|  | n=200 observations, n=68 infants | | | | | n=195 observations, n=65 infants | | | | |
| **Parameter** | **Beta** | **SE** | **95% CI** | **z** | **p-value** | **Beta** | **SE** | **95% CI** | **z** | **p-value** |
| Group |  |  |  |  |  |  |  |  |  |  |
| ABC | Ref |  |  |  |  | Ref |  |  |  |  |
| ACB | -0.12 | 0.46 | -1.02 - 0.78 | -0.26 | 0.80 | -0.37 | 0.48 | -1.31 - 0.57 | -0.77 | 0.44 |
| BAC | -1.10 | 0.46 | -2.00 - -0.20 | -2.39 | 0.017 | -1.24 | 0.46 | -2.15 - -0.33 | -2.66 | 0.008 |
| BCA | 0.14 | 0.44 | -0.72 - 1.00 | 0.31 | 0.76 | 0.00 | 0.44 | -0.87 - 0.87 | 0.00 | 1.00 |
| CAB | 0.17 | 0.46 | -0.73 - 1.07 | 0.37 | 0.71 | 0.03 | 0.46 | -0.88 - 0.95 | 0.08 | 0.94 |
| CBA | -0.33 | 0.43 | -1.17 - 0.51 | -0.77 | 0.44 | -0.42 | 0.44 | -1.29 - 0.44 | -0.96 | 0.34 |
| Treatment |  |  |  |  |  |  |  |  |  |  |
| A | Ref |  |  |  |  | Ref |  |  |  |  |
| B | -0.68 | 0.10 | -0.87 - -0.49 | -6.86 | <0.001 | -0.69 | 0.10 | -0.89 - -0.49 | -6.88 | <0.001 |
| C | -0.03 | 0.10 | -0.23 - 0.16 | -0.33 | 0.75 | -0.04 | 0.10 | -0.24 - 0.15 | -0.44 | 0.66 |
| Day |  |  |  |  |  |  |  |  |  |  |
| 1 | Ref |  |  |  |  | Ref |  |  |  |  |
| 2 | -0.01 | 0.10 | -0.20 - 0.19 | -0.07 | 0.94 | -0.02 | 0.10 | -0.22 - 0.17 | -0.25 | 0.81 |
| 3 | -0.19 | 0.10 | -0.38 - 0.01 | -1.88 | 0.060 | -0.20 | 0.10 | -0.39 - 0.00 | -1.98 | 0.048 |
| 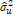Intercept | -4.86 | 0.31 | -5.47 - -4.24 | -15.49 | < 0.001 | -4.70 | 0.32 | -5.34 - -4.07 | -14.53 | < 0.001 |
| \| 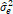 \| \| --- \| | 1.09 | 0.22 | 0.74 - 1.60 |  |  | 1.07 | 0.22 | 0.72 - 1.59 |  |  |
| \|  \| \| --- \| | 0.32 | 0.04 | 0.25 - 0.41 |  |  | 0.33 | 0.04 | 0.25 - 0.42 |  |  |

SE Standard error, CI confidence interval,
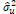
 between infant variance,
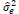
residual error

**Table S6:** Parameter estimates for the percentage of time spent with SpO2 of 88% or more using LMM on mITT and PP populations.

|  | **Modified intention-to-treat population** | | | | | **Per protocol population** | | | | |
| --- | --- | --- | --- | --- | --- | --- | --- | --- | --- | --- |
|  | n=200 observations, n=68 infants | | | | | n=195 observations, n=65 infants | | | | |
| **Parameter** | **Beta** | **SE** | **95% CI** | **z** | **p-value** | **Beta** | **SE** | **95% CI** | **z** | **p-value** |
| Group |  |  |  |  |  |  |  |  |  |  |
| ABC | Ref |  |  |  |  | Ref |  |  |  |  |
| ACB | 0.11 | 0.48 | -0.83 - 1.05 | 0.23 | 0.81 | 0.37 | 0.50 | -0.60 - 1.35 | 0.75 | 0.46 |
| BAC | 1.06 | 0.48 | 0.12 - 1.99 | 2.21 | 0.027 | 1.20 | 0.48 | 0.25 - 2.15 | 2.47 | 0.013 |
| BCA | 0.00 | 0.46 | -0.89 - 0.89 | 0.00 | 1.00 | 0.14 | 0.46 | -0.76 - 1.05 | 0.30 | 0.76 |
| CAB | -0.27 | 0.48 | -1.21 - 0.66 | -0.57 | 0.57 | -0.13 | 0.48 | -1.08 - 0.82 | -0.27 | 0.78 |
| CBA | 0.18 | 0.45 | -0.70 - 1.05 | 0.40 | 0.69 | 0.30 | 0.46 | -0.60 - 1.21 | 0.65 | 0.51 |
| Treatment |  |  |  |  |  |  |  |  |  |  |
| A | Ref |  |  |  |  | Ref |  |  |  |  |
| B | 0.61 | 0.10 | 0.42 - 0.81 | 6.28 | <0.001 | 0.63 | 0.10 | 0.43 - 0.82 | 6.34 | <0.001 |
| C | 0.00 | 0.10 | -0.19 - 0.19 | -0.02 | 0.99 | 0.01 | 0.10 | -0.18 - 0.20 | 0.10 | 0.92 |
| Day |  |  |  |  |  |  |  |  |  |  |
| 1 | Ref |  |  |  |  | Ref |  |  |  |  |
| 2 | 0.01 | 0.10 | -0.18 - 0.20 | 0.05 | 0.96 | 0.02 | 0.10 | -0.17 - 0.22 | 0.22 | 0.83 |
| 3 | 0.07 | 0.10 | -0.12 - 0.26 | 0.73 | 0.46 | 0.08 | 0.10 | -0.11 - 0.28 | 0.83 | 0.40 |
| 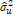Intercept | 3.58 | 0.33 | 2.94 - 4.22 | 11.01 | < 0.001 | 3.42 | 0.34 | 2.76 - 4.08 | 10.18 | < 0.001 |
| \| 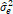 \| \| --- \| | 1.19 | 0.23 | 0.81 - 1.75 |  |  | 1.17 | 0.24 | 0.79 - 1.74 |  |  |
| \|  \| \| --- \| | 0.31 | 0.04 | 0.25 - 0.40 |  |  | 0.32 | 0.04 | 0.25 - 0.41 |  |  |
